# Analysis of policy responses to COVID-19: a case study in Babol University of Medical Sciences (BUMS), Iran

**DOI:** 10.1186/s12962-022-00404-w

**Published:** 2022-12-12

**Authors:** Zeynab Farhadi, Morteza Salemi, Mohammad Ali Jahani

**Affiliations:** 1 Social Determinants of Health Research Center, Health Research Institute, Babol University of Medical Sciences, Babol, Islamic Republic of Iran; 2 Social Determinants in Health Promotion Research Center, Hormozgan Health Institute, Hormozgan University of Medical Sciences, Bandar Abbas, Islamic Republic of Iran

**Keywords:** COVID-19 pandemic, Health policy, Policy making, Disease outbreaks

## Abstract

**Background:**

Preparation and financing of treatments, control of disease by limited resources, are known as the most important challenges encountered by the policy-makers involved in an epidemic outbreak. Therefore, the present study was conducted to analyze the policy responses of Babol University of Medical Sciences (BUMS) to Coronavirus (COVID-19).

**Methods:**

A qualitative study was performed to investigate the policy responses of BUMS to COVID-19 in Babol of January to March, 2021. The statistical population included the experts, pundits, policy-makers and planners involved in four areas of management, treatment, healthcare, and health donation. Data collection was done according to interviews and policy documents, and the obtained data were analyzed based on the Walt and Gilson’s policy triangle.

**Results:**

There are five main themes to names: policy context, policy analysis, policy-making process, actors and stakeholders and 16 sub-themes. After several rounds of revision, the text of the interviews and policy documents were tagged and finally, various issues related to sub-themes were extracted. Also, two sub-themes entitled (improving the policy framework, People’s participation) were obtained from the strategies to reduce the incidence of Covid-19 theme.

**Conclusions:**

(BUMS) was able to use the capacities and skills of experienced physicians, specialists and nurses to respond to patients awaiting treatment. Therefore, most of the policies were aimed at patient care and treatment. The lack of financial resources was compensated by health donors. But the (BUMS) could not use the power of the city government to control traffic and comply with health protocols and prevent infections. It was mainly the formulation and implementation of irregular and unstable policies.

## Introduction

The official CDC data from China showed that the COVID-19 pandemic could cause more infection and death compared to SARS or MERS, and most of the infected people, without any symptom or with mild symptoms, were able to release the virus and transmit it to others. Therefore, preventing the spread of COVID-19 is highly challenging and requires a strict monitoring [1]. It is noteworthy that the therapeutic strategies to combat the infection have only been of supportive types and prevention has proved to be the best weapon to reduce transmission in the society [2].

To control the COVID-19 pandemic, the current world leaders have sought to implement the best practice using a variety of strategies [3] and take the proper countermeasures to reduce the destructive effects of COVID-19 [2]. In Iran, after the diagnosis of the first case of COVID-19 on February 19, 2020 in Qom, despite the heavy political sanctions against providing the medical equipment and life-saving medications to deal with emergencies, implementation of the appropriate policies was put on the agenda as a general principle [1–5]. Therefore, as in other countries, it was attempted to increase the capacities by strengthening the primary healthcare and social supports [6] and prioritizing the actions of the health system in order to make the best use of the available resources in Iran [5]. In such cases, the most important challenges encountered by the policy-makers are making preparations and providing finance for the outbreak in a complex political environment with limited resources, balancing the investments in public health and health services, creating the capacity for research and development concerning the outbreak, and prevention and control of infection [7]. By tackling these challenges, the policy-makers would be able to establish the policies required for confronting the social problems and solve the problems caused by the previously adopted policies [8].

Djalante et al. conducted a study of policy science analysis in ASEAN (Association of South East Asian Nation). The purpose of this study was how political science can help legislators and policymakers understand the response to the Covid-19 pandemic. There were similarities and differences in the response to the Covid-19 crisis and its management in Southeast ASEAN countries. For example, in Indonesia, a strategic response was received from the president; this is while it was received from the Prime Minister in Malaysia and Singapore. Operational levels in countries such as Indonesia and Laos were implemented by the task force. Countries like Indonesia and the Philippines could not comply with strict quarantine due to poverty [9]. Another study by Kavaliunas and et al. was conducted with the aim of describe and analyze the approach of the health care system, Policy overview, actions and consequences of the Swedish country combating the pandemic of Covid-19 [10]. Also, a study was conducted under the title of policy making in the Covid-19 crisis and the impact of its consequences on the political and economic performance of the society with the approach of humanities in Iran [11]. But no study was conducted on policy analysis in the field of health systems; we decided to Analysis of Policy Responses to COVID -19 at Babol University of Medical Sciences.

Babol is a university city located in Mazandaran province (northern Iran), 400 km from Qom city, between the Caspian Sea and the Alborz mountain range, at distances of 15 and 211 km from the Caspian Sea and Tehran (the capital of Iran) respectively. The city, due to its temperate climate, green forests, and proximity to the Caspian Sea, is considered as a tourist-friendly city and the second home for Tehran dwellers. One of the most important religious cities in the north of Mazandaran province. Since the cities in this province are close to each other and contain many transit routes, these cities usually face a heavy daily traffic. Babol is known as the first city of Mazandaran province holding a Medical Sciences university since 1983. Therefore, a large number of medicine specialists are currently working in various fields of medical sciences in this city. Due to the similarity of religious factors, there is a lot of traffic between the two cities of Qom and Babil. Thus, Babol was declared the second city of the Red Zone less than two days after the first city. Due to having several university hospitals, this city has hosted many patients from the nearby cities. The (BUMS) was among the first universities to suffer from the crisis in the early days of the virus entering the country. The (BUMS) includes six teaching hospitals, two private hospitals, over 360 faculty members, ICU specialists, lung specialists, internal medicine specialists, several infectious disease specialists, pharmacists, environmental health specialists, epidemiologists, and experienced nursing staff, with “non-invasive” trainings and 15 years of work experience, who could substantially support the services. People became infected, and since it was the first appearance of the COVID-19 crisis in the country and the resources were limited, everything was ambiguous for the people and the (BUMS) medical staff at first. At that time, there was no strong evidence against COVID-19, and no specific regulations and guidelines had been formulated for it. The first instructions from the Ministry of Health were received on February 23 (BUMS) [12–16]. COVID-19 has posed several challenges to the (BUMS) from the very beginning. At first, the policy of (BUMS) was to choose the referral hospital and accept all the patients referred to the medical centers. Then, the establishment of the university crisis headquarters was the maximum use of the scientific capacity of faculty members, city officials and donors. Given that there was no experience of coping with COVID-19 in the country, Hence, given the role of policy-making and its impact on cost control and success or failure of the health systems, scientific and systematic policy analyses are essential [17]. Policy analysis can provide a clear picture of how and why the effective policies are developed and implemented over time [18]. Therefore, the present study was conducted to analyze the policy responses the (BUMS) to COVID-19 from January to March, 2021.

## Methods

**Fig. 1:**
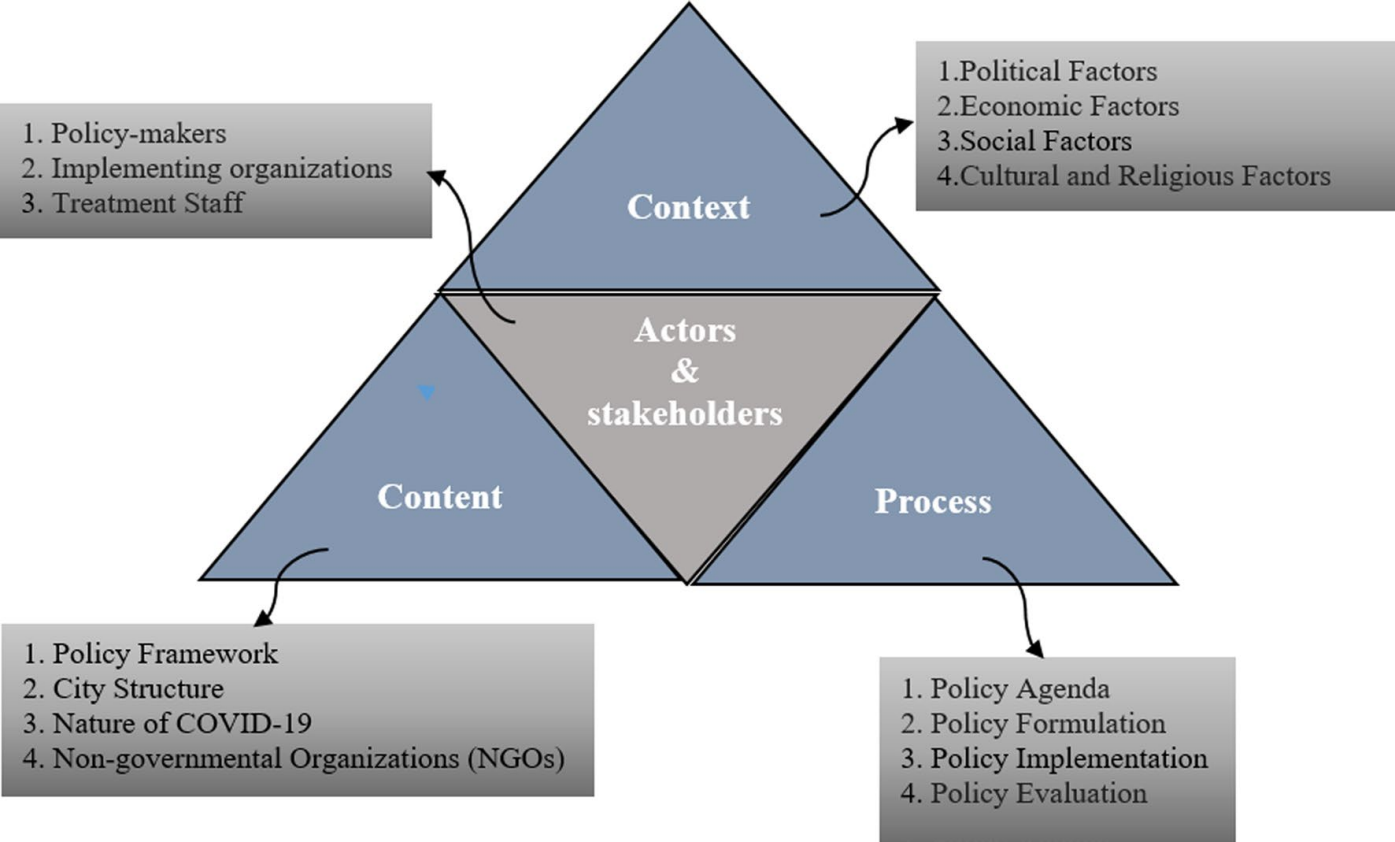
The Walt and Gilson’s policy triangle framework

A retrospective qualitative study was conducted to explore the policy responses the (BUMS) to COVID-19 from the beginning of January to the end of March, 2021 in Babol (Iran). The Walt and Gilson’s framework can help researchers in systematic understanding and analysis of the health-related policies. This framework consists of four elements: context (why), content (what), process (how), and actors (who) (Fig. 1). Walt and Gilson argue that health policy-making is an interactive process in a specific social, economic, and cultural context in which actors are at the center of the process [19]. The research approach was deductive and seeks answers to the following general questions:

1. Was the (BUMS) able to respond to the patients waiting for hospitalization? 2) Was the (BUMS) able to use the city's governing power to quarantine and comply with health protocols?
2. Were there experienced doctors, specialists and nurses at (BUMS) to treat and care for patients with COVID-19?
3. Was the the (BUMS) able to make the right connection between the health and care system?
4. Was the (BUMS) able to take full advantage of the power of doctors and specialists affiliated with the university?
5. Was the (BUMS) able to overcome the limitations of financial resources?
6. Were the policies and programs of the (BUMS) sustainable?

## Method structured including: data collection, data analysis, conformability and transferability

### Data collection

The data was mainly collected through interviews. But policy documents (laws, regulations, meeting minutes and related flowcharts) were among the other methods used to collect data; In order to complete the data and lack of access to some key people (stakeholders), so of the comments in the minutes of the meetings were used. The interviews were conducted after ethical approval and obtaining the ethics code No. 724133083 and a letter of introduction from the Deputy of Research and Technology of the university. Finally, the interviews and the policy documents (by laws, procedures, instructions, minutes and flowcharts) were analyzed [20].

### Data analysis

To analyze the data, the text of the interviews was studied several times in order to gain a comprehensive understanding of them. All the interviews and decisions of the COVID-19 crisis committee were analyzed and coded as units of analysis. Paragraphs and sentences or words were also considered as semantic units. Then, the semantic units were abstracted, conceptualized and coded according to their hidden concepts. The codes were compared with each other in terms of their similarity and difference and classified into more abstract classes using specific labels the Walt and Gilson’s framework [21]. The interview guide was designed according to the four elements of Walt and Gilson framework. In designing the interview, sub-themes policy context, including (Political, Economic, Social Cultural and Religious factors), sub-themes policy content including (policy framework, city structure, nature of COVID-19 and Non-governmental Organizations (NGOs)), sub-themes policy process including (policy agenda, formulation, implementation and evaluation) and sub-themes actors and stakeholders including (policy-makers, implementing organizations, people and treatment staff) were considered. At the end, the participants were asked about the Strategies to reduce the incidence of Covid-19.

### Conformability

The revision technique was used to increase the validity of the data. For this purpose, at the end of each interview, the participant was provided with a summary of his/her statements to ensure that they were understood and written correctly. Understanding of the textual data can lead to drawing a valid inference from the data related to the Research questions [21].

### Transferability

The statistical population of the present study included, experts, pundits, policy-makers and planners (BUMS). Inclusion criteria for participating in the interview included the following: (1) Participants who worked in the areas of management and administration, treatment, health care and health donation; were chosen. (2) Managerial and executive participants attended at least 15 sessions of the COVID-19 Crisis Committee in the (BUMS) and participants who did not cooperate in formulating and implementing of COVID-19 coping programs and policies were excluded from the criteria (Table 1). Furthermore, to increase the transferability of the findings, it was attempted to select the appropriate samples at different levels of decision-making and implementation and to collect and analyze the data simultaneously [22]. To maintain the privacy, all the participants were mentioned based on their specialization.

**Table 1:** Expertise of the participants in the study

Area	Expertise	Number of interviewees	Area	Expertise	Number of interviewees
Management	Internal medicine physician	3	Treatment	Virologist	2
	Infectious disease specialist	1		Neonatal physician	1
	Environmental Health specialist	1		Pulmonologist	3
	Pharmacist	1		ICU specialist	2
				Anesthesiologist	2
				Infectious disease specialist	2
				Environmental Health specialist	2
				Epidemiologist	1
Treatment	Infectious disease specialist	4	Health donors		3
	Cardiologist	3			
	Nephrologist	1			
Total					32

### Interview method

The questions were designed to reflect the views of the experts, pundits, policy-makers and planners involved in tackling COVID-19. Initially, the purpose of the study was explained to the selected individuals by telephone or verbal. If they agreed to participate in the interview, for the first three interviews, the interview guide was emailed and then the interview was conducted, but after that, the interview guide was not sent due to performance bias control. All the interviews were conducted face-to-face by a researcher [23]. In case that the conversation was not allowed to be recorded, notes were taken during the interview. Out of 32 interviews, only one interview was not allowed to be recorded and it was done through taking the notes. A time range of 35–90 min was selected for the interviews. At the end of each interview, the researcher encouraged the participants to freely express their feelings by asking them if they wanted to add anything else to their views. The interviews continued until reaching the data saturation stage [24].

### The context of Babol city and health system in Iran

Babol has a population of 530,000, and in terms of economy and income, it is higher than the average income of the surrounding cities. In terms of forming volunteer and public groups and helping people in need, it has a very good participation; but in terms of political participation, it is relatively low [25]. As an important point, principle 29 of the Constitution of the Islamic Republic of Iran emphasizes that every Iranian has the right to enjoy the highest attainable level of health. The Ministry of Health, Treatment and Medical Education is obliged to realize this goal by designing and implementing health policy at the national level. The Ministry of Health, Treatment and Medical Education assigns its implementation to universities of medical sciences across the country. There is at least one medical university in every province. The President of the University of Medical Sciences is the highest health official of the province who reports to the Minister of Health, Treatment and Medical Education and is responsible for public health, providing health care in public facilities and medical education [26].

## Results

As explained in the method, according to the Walt and Gilson’s policy triangle framework, there are 5 main themes and 16 sub-themes. After several rounds of revision, the text of the interviews and policy documents were tagged and finally, various issues related to sub-themes were extracted. Also, two sub-themes entitled (improving the policy framework, People’s participation) were obtained from the strategies to reduce the incidence of Covid-19 theme. Themes and sub-themes based on the Walt and Gilson policy triangle framework are shown in the table below (Table 2).

**Table 2:** Themes and sub-themes based on the Walt and Gilson policy triangle framework

Theme	Subtheme	Theme	Subtheme	Theme	Subtheme
Policy context	Political factors	Policy content	Policy framework	Policy process	Policy agenda
	Economic factors		City structure		Policy formulation
	Social factors		nature of COVID-19		Policy implementation
	Cultural and religious factors		Non-governmental Organizations (NGOs)		Policy evaluation
Actors and stakeholders	Policy-makers	Strategies to reduce the incidence of Covid-19	Improving the policy framework		
	Implementing organizations		People’s participation		
	people				
	treatment staff				

### Policy context

**Fig. 2:**
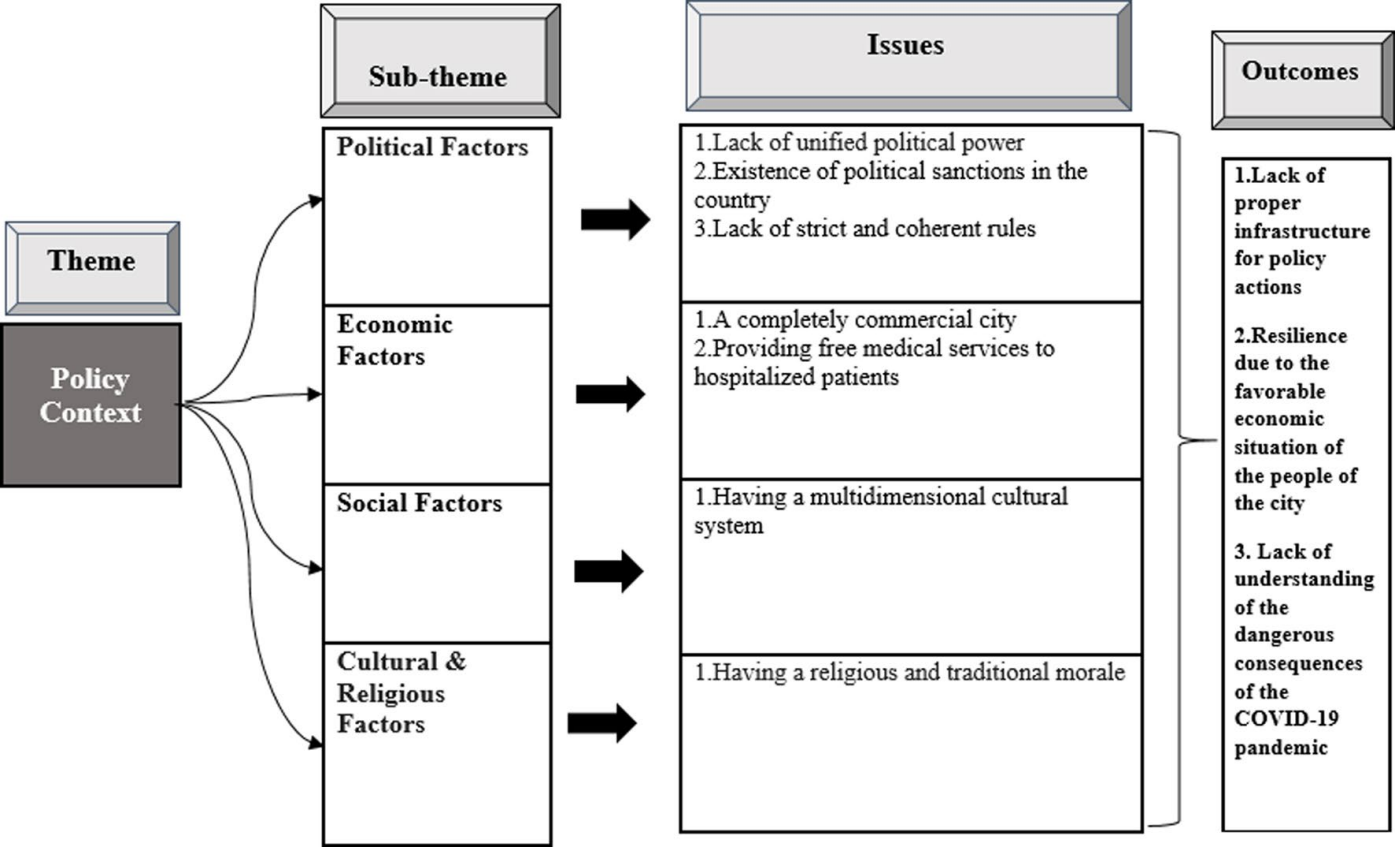
Issues and outcomes of the policy context analysis

Policy context findings showed that the lack of a suitable platform for administrative action and implementation of infrastructure and enforcement requirements was the most important factor hindering the full implementation of policy responses (BUMS) to COVID-19. The outcomes of policy context analysis are shown in (Fig. 2).

#### Political factors

Analysis of interview data and documents showed that the rules were not so strict and coherent; because the low-income sections of society should not be harmed. Restrictions on health protocols were low and unstable. One of the interviewees said:

I think there was no potential for exerting legal actions by the monitoring and prevention systems. Therefore, this part of the monitoring job was not correctly rendered at the society. They did not define a specific mechanism, and did not enact a written law for that matter. (Internal specialist).

#### Economic factors

Most of the people in the study area make their living by trading. Hence, the people had a good financial condition, which is why in the first phase of the crisis, when the city was suddenly stricken by the COVID-19 crisis, they had a good resilience.

Babol is the economic center of Mazandaran province, which was formerly called “Barforoush” (seller) meaning that it was a hub for most of the economic activities in the province. People are rich and we should not compare them with the people in other provinces. In this way, they contributed to providing the personal protective equipment which was necessary for patients and medical staff. (Infectious disease specialist).

Although the medicines used for the treatment of the hospitalized patients with COVID-19 were very expensive, they were completely free for the patients.

#### Social factors

The experts believed that the social factors in Babol was not based on a monoculture or one-dimensional system, and it rather contained the multicultural individuals with multiple subcultures. For this reason, the reference points were culturally and socially diverse. One of the experts admitted:

We have a system which contains multiple subcultures and a multifaceted cultural system in which the people have different gathering places. Today they go to a wedding or mosque and tomorrow they go to the stadium. (Anesthesiologist).

Due to having multiple social factors, the participation of people in the community in observing health protocols was difficult.

#### Cultural and religious factors

Culturally, gathering of people is very common in the study area. Since the whole city were to celebrate the Persian New Year (Nowruz), it was difficult to keep them under control. Furthermore, due to the people’s religious beliefs, many religious gatherings and ceremonies were held in the city. Nevertheless, these gatherings and ceremonies were changed to some extent, as some of the interviewees said:

Religious groups, even the theology students, came to the hospital and took part in transporting the patient and oxygen. The religious groups came and allocated the budgets of their religious gatherings to fulfill the needs of the patients with COVID-19, and we were able to buy four suction devices. (Internal specialist).

Actually, one of the good measures taken by the COVID-19 crisis committee at the county and provincial levels was making decision equally for religious and non-religious institutions, sport centers and markets. (environmental health specialist).

The people noticed the situation, but the general atmosphere and the false news misled them and potentially encouraged them not to observe the protocols as expected. The public media and cyberspace, in some cases, sent the message that things were not that serious. The WHO itself sent the big wrong message that the face mask had not enough coverage and it should be used only in medical centers but not in the public. This contradiction made us experience several pandemic peaks. (infectious disease specialist).

However, some people in the community still did not believe in the epidemic. Despite the deaths, COVID-19 seemed simple and transient to them.

### Policy content

**Fig. 3:**
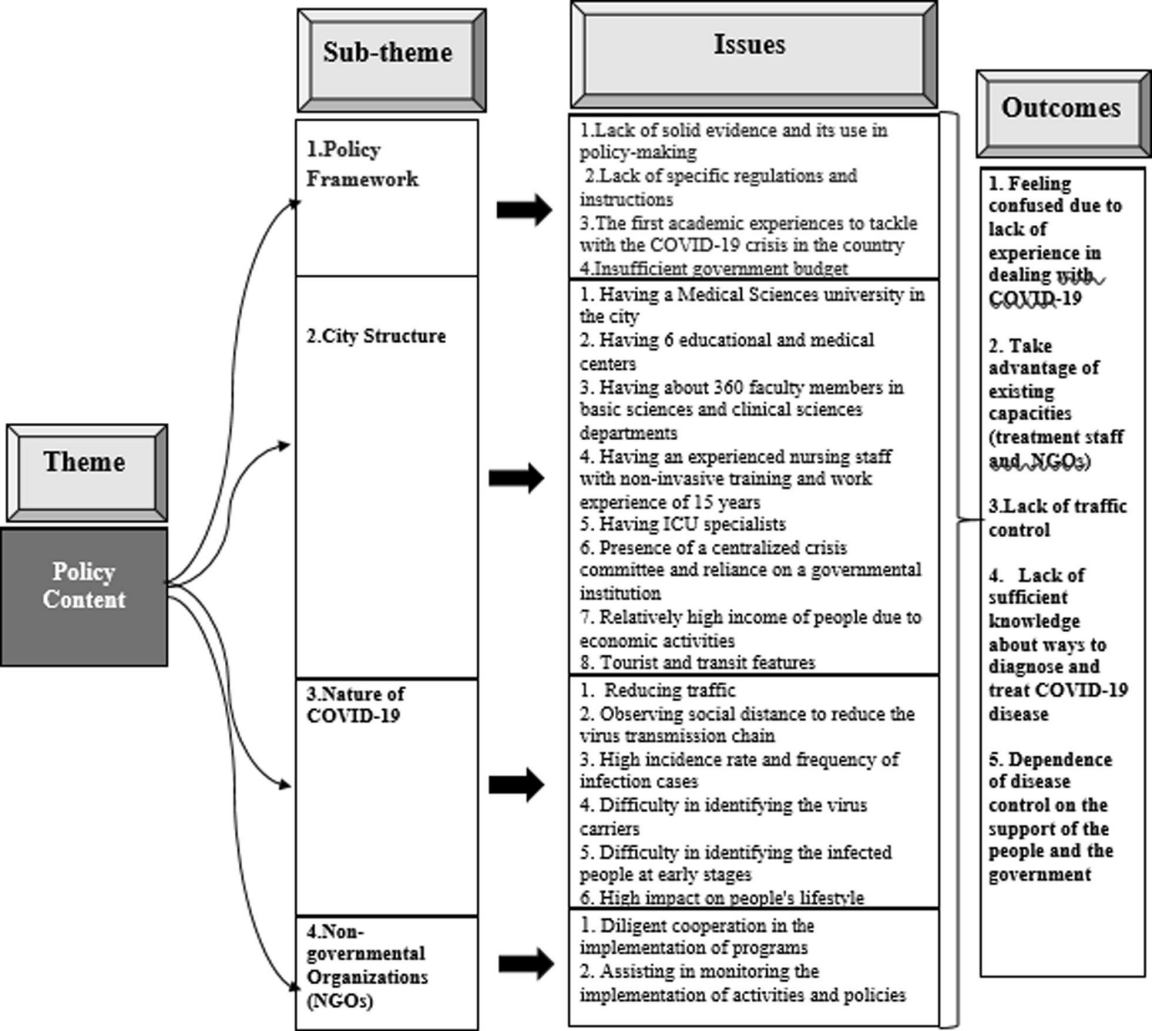
Issues and outcomes of the policy content analysis

The policy content clarifies that: Which policy is better? Which health services should be omitted? Which policy tools are needed? Who makes and implements the decisions? [29]. Policy analysis is designed to better understand the policy process and to provide the policy-related knowledge and evidence on economic and social problems. The information produced in this way can be used in the policy-making process to solve the policy problems [30]. The outcomes of policy content analysis are shown in (Fig. 3).

#### Policy framework

The University officials were could rapidly overcome their psychological limitations, and the COVID-19 crisis committee was formed in the university. This committee was hold at the presence of the supply council, the city governor and the medical staff, in which the prevention and treatment policies were adopted. To prevent the spread of the disease, the university asked for help of the authorities governing the city. Since, at that time, the people living in the city were to celebrate Nowruz and many local markets were open, the COVID-19 crisis committee with the help of the supply council could prevent the opening of the market. One of the interviewees said:

Another strength of the university was to engage various executive departments of the city, even the district governors, Basij, theology students, and anyone we thought could help us in this epidemic. We engaged everyone. The markets were closed, even at the last days of the year, when people normally go shopping for the New Year, the market was actually closed. A large number of public places were sealed by the health center and the police. If these measures were not taken, we would face unprecedented casualties in the first peak. (Environmental health specialist).

Given that (BUMS) was the first university in the country to encounter a pandemic. At first, it was difficult to design clear and transparent programs and policies.

#### City structure

The people with high income and the good behavioral process adopted by the university committee to govern the hospitals were among the privileges of Babol. Another feature of Babol is its transit route. Since the cities of Mazandaran province are very close to each other, many people commute daily from other cities to this city to attend their workplace or go shopping. Another feature of the city is its pleasant weather, which attracts many people from south and east of Iran. Significantly, entrance of the citizens of Tehran due to the two-week shutdown at the first peak of COVID-19 was one of the major issues that increased the traffic in the city.

#### Nature of the COVID-19 virus

The interviewees acknowledged that, during the past 10–15 years, the policies of the university have focused on non-infectious diseases such as cancer and cardiovascular disease. In fact, they were unaware of some types of infectious disease because they did not lead to considerable mortality. The only common infectious disease was the H1N1 flu which was simply controlled. The older versions of COVID-19, namely SARS-CoV and MERS-CoV, practically did not affect Iran or they had a very low incidence. Therefore, they did not have any related experiences, in terms of either laboratory or imaging. However, the nature of the COVID-19 virus showed that it could not be controlled only by the medical staff alone. In fact, it was necessary to reduce the traffic, set up home isolation in case of infection, and observes the social distance to break the chain of infection. The virus had a great impact on people's lives due to its high incidence, high frequency of the infected cases, and unidentifiability of the carriers. One of the interviewees stated:

To succeed in controlling the virus, we should consider three items: the triple-T strategy (which stands for targeting, testing, and tracking), contact tracing, and treatment. The (BUMS) did very well in testing and treatment from the beginning of the fight against COVID-19. We launched the PCR diagnostic test laboratory on March 7, 2020. This means that we set up this laboratory much earlier than other medical sciences universities in the country. Nevertheless, the most important issue was contact tracing which was not properly performed neither in the (BUMS) nor in other parts of Iran. (virologist).

After one week, the statistics rose to an extent that, for example, we had 50 to 60 inpatients a day, occupying all the hospital beds in three days. Therefore, we needed to establish another hospital. This amount of inpatients in such a short period had not been seen in Iran or any other country in the world. Our hospitalization rate even reached 110 patients a day. (infectious disease specialist).

Considering the place where the first case of COVID-19 appeared and the points where it unofficially spread in the country, the interviewees stated that the researchers had not given them any information. However, according to the official declarations, the first chains of infection started from Qom, Tehran, Gilan and Babol. All these events occurred in our country within two weeks. In other words, Babol was among the cities which were affected by the crisis in the early days of the virus arrival in the country, and its citizens were infected.

#### NGOs and donors in the city

Experts acknowledged that the Babol Health Charity Foundation, a voluntary non-governmental organization, played an important role during the COVID-19 crisis and covered about one-seventeenth of the treatment costs. This foundation, as a link between donors and the university, acted systematically and played a very important role in providing personal protective equipment, pulse oximeter, oxygen generator, CPAP, Bipap and even the food required for the medical staff. One of the interviewees expressed:

The donors provided us with very good protections. In the turbulent situation in March, the donors helped the patients by providing them with medicines. IVIG cost at least $ 2,000, which was later shown to have little effect, without being sure about its efficiency. Meanwhile, the donors were really determined and stayed with us at the hospital, and thereby we could manage the situation in that crucial condition. (infectious disease specialist).

### Policy process

**Fig. 4:**
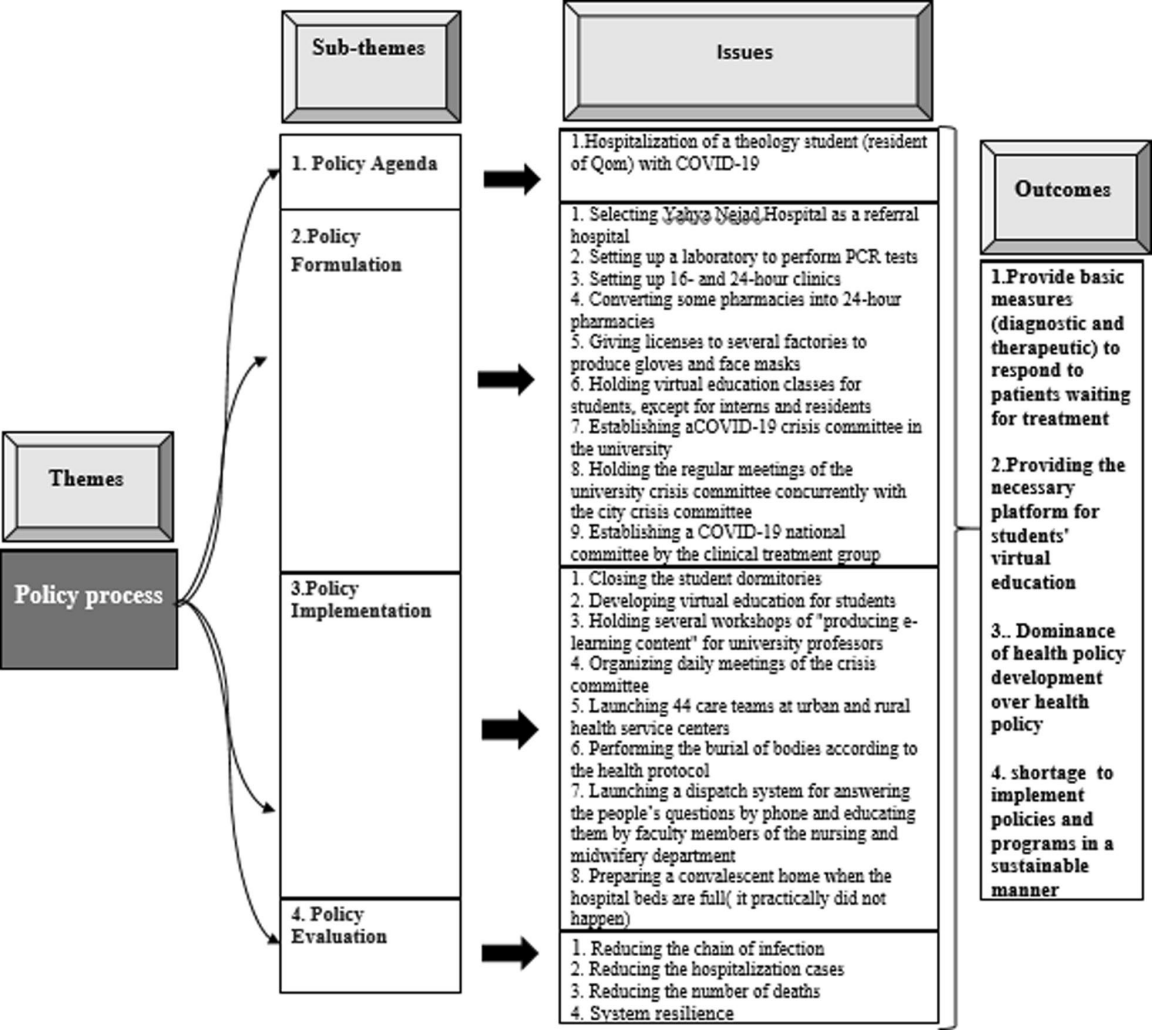
Issues and outcomes of the policy process analysis

Policy-making is a process of formulation, implementation and evaluation of the different decisions and policies adopted by official and unofficial decision-makers and has direct and indirect effects on people’s lives [31]. The outcomes of policy process analysis are shown in (Fig. 4).

#### The policy agenda of COVID-19 crisis

All the participants acknowledged that the crisis code was announced in Babol after hospitalization of a theology student from Qom, who had symptoms of COVID-19. One of the interviewees explained:

For the first time, the infectious diseases resident reported a patient with critical condition, who was a theology student from Qom. At the same time, the disease spread in Qom. I checked his CT scan and found that they were compatible with his symptoms. At the beginning, the infected cases were two to three persons a day, but they significantly increased within a week, due to some factors such as social gatherings. (Infectious disease specialist).

Therefore, the crisis code was announced by the (BUMS) in Babol on February 20, 2021.

#### Policy formulation

To deal with COVID-19 in terms of reducing the incidence and treatment of the hospitalized patients, the (BUMS) established the COVID-19 crisis committee. The COVID-19 crisis committee meetings were held daily during the first peak of the crisis. According to the instructions of the Ministry of Health, one of the hospitals was assigned as a referral hospital for the patients with COVID-19. Since Yahya Nejad Hospital had been previously used for the patients with H1N1 flu, it was selected for this purpose. However, all the beds of Yahya Nejad hospital become full within 3 days and inevitably a section of Rouhani hospital was dedicated to the patients with COVID-19. To identify the patients before they reach an acute stage, they were required to be diagnosed in early stages. Therefore, the COVID-19 crisis committee decided to use one of the university laboratories for PCR diagnostic test and establish 16- and 24-h clinics. A private laboratory and a round-the-clock radiology center were also employed to help the governmental sector. To prevent overcrowding in pharmacies on night shifts, some pharmacies were allowed to be open around the clock. Moreover, some factories of the city were licensed to produce adequate personal protective equipment, such as gloves, face masks, and disinfectants, to meet the demands of the people. A lesson of the students, except for medical interns and residents, was taught using the virtual education tools.

#### Policy implementation

Student dormitories were closed to control the disease, and several “virtual education content production” workshops were held for university professors. 44 care teams were organized at the urban and rural health service centers. Burials were done according to the protocol. A dispatch system was used to answer the people’s questions and train them with the help of the university faculty members. Moreover, some volunteers were train to support the hospital. A convalescent home was put on standby to be used when the hospital beds were full. One of the interviewees said:

We used to move the crisis committee to the target places which had the infection. That is, the crisis committee was called the crisis committee of the university. This trend continued until the crisis in the city reached a stable condition and the methods for dealing with the disease reached a mature level. Since then, the meetings of the crisis committee were held at the university. (Internal medicine specialist).

As of March 5, there was no specific national or global protocol to combat COVID-19. One of the interviewees added:

At first, there was a lot of confusion about choosing the right protocol when the COVID-19 pandemic started. Everyone was writing his own prescription and it was confusing. At early stages, medications had to be taken based on clinical trials. There was not enough time to test them on patients. We gave the patients Kaletra, Tamiflu, Hydroxychloroquine, IVIG, Plasma therapy and whatever we could, because we were really confused. At first, a very primitive national protocol was issued that provided a brief guide for professors and those involved in COVID-19 on how to manage the disease. It was like a roadmap, but it was not what we really needed. Since drug interactions were not taken into account, we had a high rate of mortality due to giving multiple drugs. Maybe it was because of the virus itself, or the prescribed medications or even the patients who used to attend the hospital at the final stages of the disease. Mortality was very high in late February and early March. (lung specialist).

Therefore, members of the subspecialty board (ICU, lung and internal medicine) and infectious disease specialists at Rouhani hospital came together to integrate the treatment and formed the national COVID-19 crisis committee.

#### Policy evaluation

Analysis of the findings showed that the (BUMS), aided by the city authorities and the volunteered people, could significantly reduce the rates of hospitalization, positive PCR and mortality by implementing the policies for fighting COVID-19 for two months (from February 20 to April 20). This condition continued for one month, the positive PCR samples were largely reduced and even reached zero in some days. At this time, the people’s fear of the epidemic was reduced. Due to certain problems associated with quarantine in the country, the health protocols were loosely followed. Most of the people concluded that the epidemic was controlled. Some deficiencies in commitment to prevention were also observed in some governmental institutions and in the (BUMS) in following the formulated policies. Opening of wedding venues, schools and religious institutions was a sign of neglecting the social distance. This led to the second peak. One of the interviewees believed:

One of our weaknesses is that we work well under maximum pressure. I think, under medium to low pressures, our procrastination emerges and our weaknesses become more apparent. (Internal medicine specialist).

**Fig. 5:**
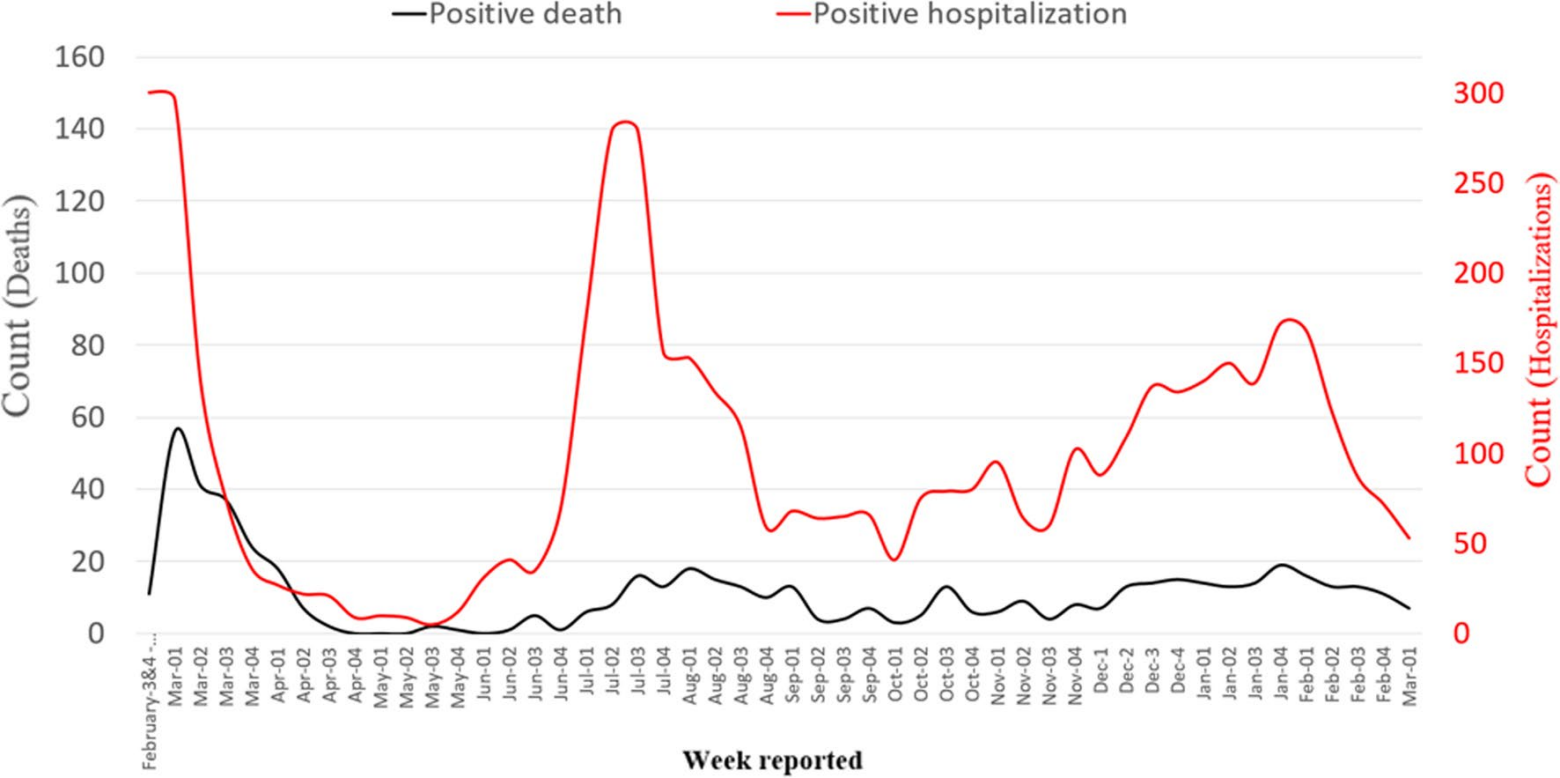
The trend of hospitalization cases, positive PCR and mortality in Babol from February 20, 2020 to March 7, 2021

From the third week of May, the number of hospitalizations raised to even more than the number of hospitalizations in the first peak, and reached the highest level in the third week of July (Fig. 5). However, the rate of mortality was lower compared to the first peak. In this regard, one of the experts admitted:

The most important reason was mutation of the virus, which strengthened its infectivity and reduced the severity of the disease at the same time. Our fears about giving a large amount of drugs to the patient and our doubt about the effectiveness of drugs had been greatly reduced. The significantly increased experience of the health system led to the reduction of the mortality rates. The hospitals were more prepared, the ICUs were much better equipped, and the issues related to oxygen and other problems in the hospitals were almost eliminated. (Infectious disease specialist).

Despite all the problems, (BUMS) was able to control the first peak, but due to the lack of continuation of the program and policies as before, caused it to experience the second peak in the third week of July 2021.

### Policy actors and stakeholders

**Fig. 6:**
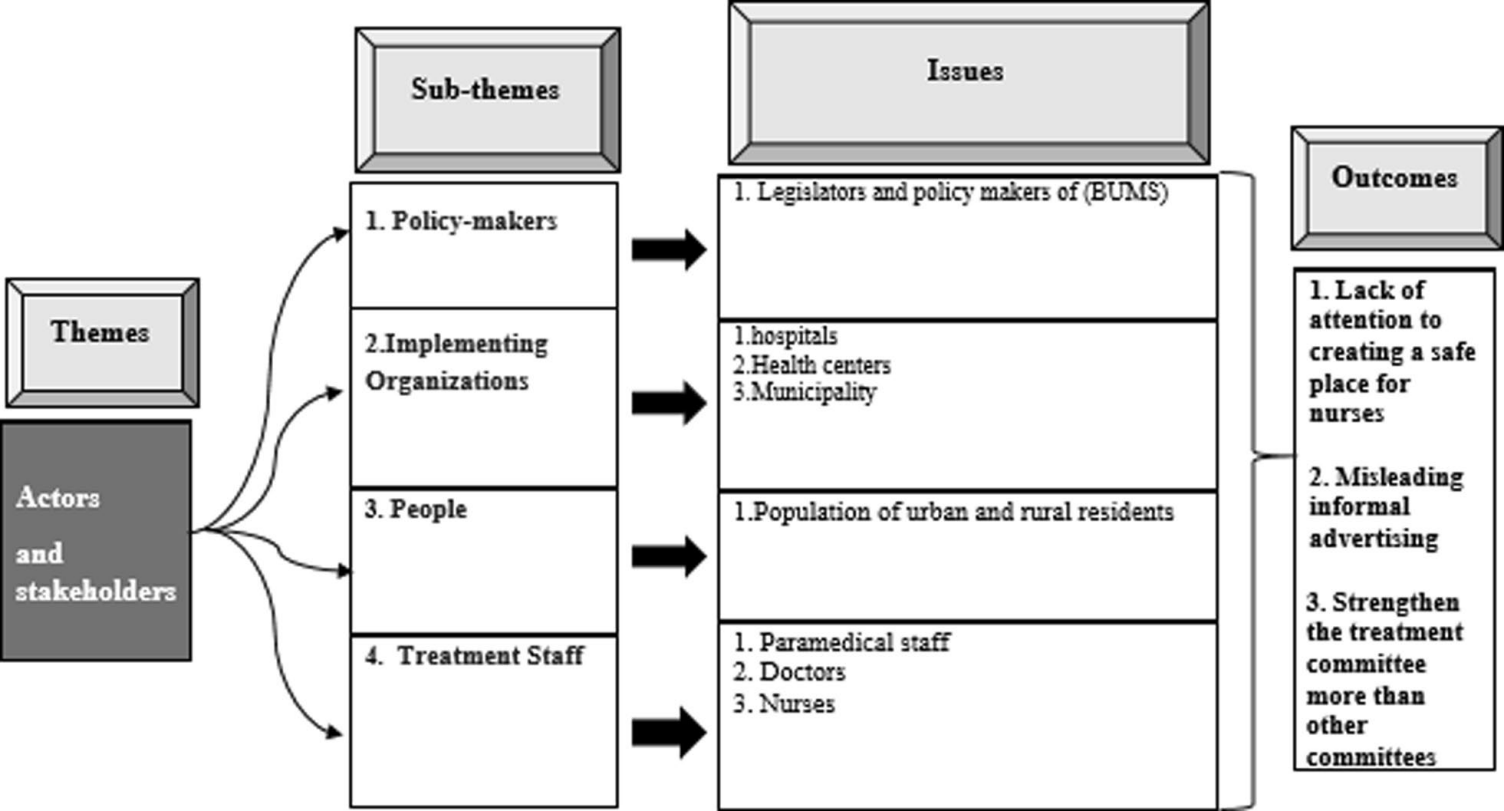
Issues and outcomes of the policy actors and stakeholders analysis

The outcomes of Policy actors and stakeholders analysis are shown in (Fig. 6).

#### Policy-makers

After hospitalization of a patient with symptoms of COVID-19 in one of the hospitals, the COVID-19 crisis committee was formed in the (BUMS). This committee consisted five working groups as care and treatment, prevention and health, epidemiology, support, and public relations working groups. Each working group had specific tasks.

The only working group which permanently performed its duty, boarded the boat, took the oar, and set off was the care and treatment working group. (Internal medicine specialist).

#### Administrative organizations

As an administrative organization, the (BUMS) mainly focused on care and treatment of the patients with COVID-19, while the city organization bodies focused on preventive policies such as controlling the traffic and gatherings and spraying the disinfectants. But the preventive organizations in the implementation could not keep pace with the (BUMS).

#### People

Since spread of the virus has a direct relationship with social distancing and lower traffic, the people can be considered as one of the main factors in controlling the COVID-19 virus. One of the experts confirmed:

Unofficial propaganda supported the belief that it would be better for a person with primary symptoms not to go to the hospital. Therefore, long delays in going to the hospital made the treatment more difficult for doctors. (ICU specialist).

#### Medical staff

Safety of the medical staff, particularly nurses, had an important role in controlling the COVID-19 virus. If nurses were infected with COVID-19, their quarantine for two weeks and substitution of their shifts would impose many difficulties to the directors of the hospitals.

Knowing that more than 80% of the medical care process is done by nurses, at the beginning of the pandemic, many of them were afflicted with severe anxiety and obsessive–compulsive disorder. The next problem was their breakroom which was not standard. Under these circumstances, if a nurse became infected, he/she could infect others in a short time. About 75% of the nurses were infected, but fortunately we did not have any mortality among the nurses. (Infectious disease specialist).

### Strategies to reduce the number of patients with COVID-19

**Fig. 7:**
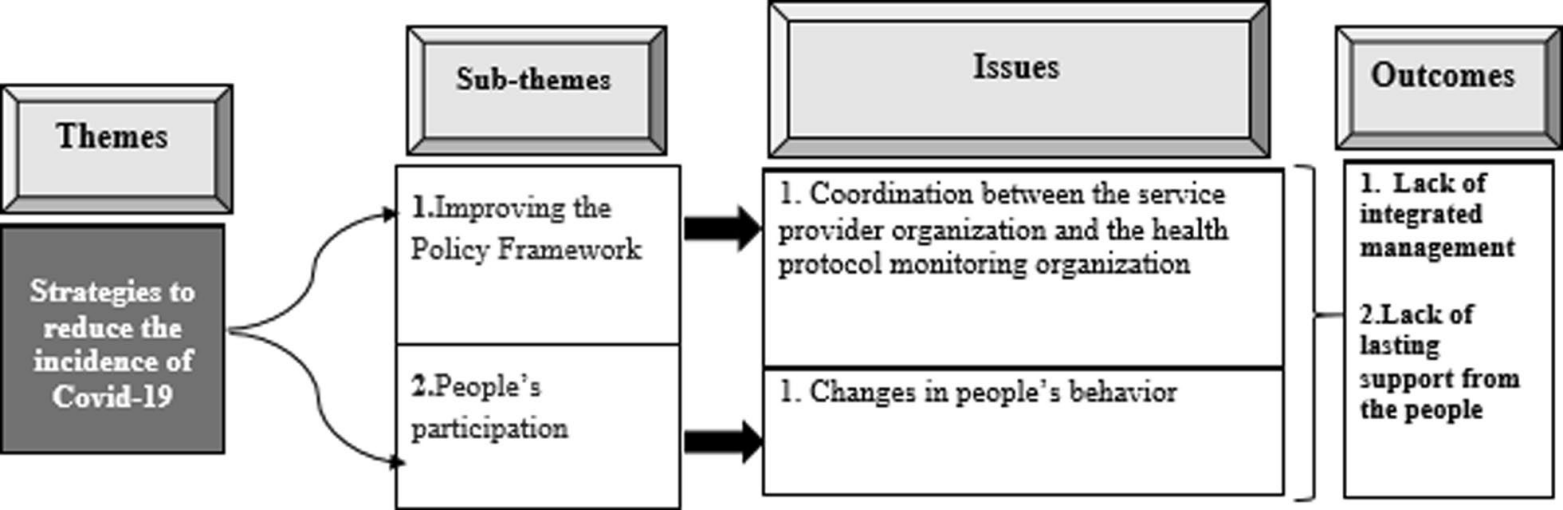
Issues and outcomes of the strategies to reduce the number of patients with COVID-19 analysis

The experts believed that tackling COVID-19 required an integrated management to coordinate the service providers and related institutions. The outcomes of Strategies to reduce the number of patients with COVID-19 are shown in (Fig. 7).

#### Improving the policy framework

Normally, the (BUMS) shall not be responsible for infection control in the city, and hospital should not be the main place for fighting the disease. In fact, all the institutions which have power must be responsible for dealing with COVID-19.

#### People’s participation in observing the protocols

People’s participation is the most important factor in defeating COVID-19, and they can break the chain by changing their behavior such as reducing their interpersonal relationships and avoiding the collective activities and gatherings.

## Discussion

With identification and registration of the first COVID-19 patient in Babol, it was officially included in the COVID-19 regions of the country on February 20, 2020 [4]. Due to the fact that the outbreak of the disease was very unexpected, the infodemiology indices failed to enhance the readiness of health care systems in the study area [32]. At first, the atmosphere was very stressful and everyone needed to be mentally and emotionally strengthened [33]. Health promotion and disease prevention require the actions of different levels of the society. Normally, the government and other institutions and organizations are responsible to take such actions. The factors related to health functions in the society have turned the health policy analysis into an important issue. Thus, the policy analysis should find some ways to simplify the situation for policy analysis in the health system. This involves formulation of a theoretical framework that can determine the significant and insignificant factors and relationships [30]. The important related factors were: overcoming the limitation of financial resources, providing the necessary facilities to deal with COVID-19, informing the people and their participation in adhering to the protocols, overcoming the psychological limitations, recognizing the disease, and discovering the methods of treatment. In the given conditions, it was necessary to postpone the development plans of the university and temporarily ignore the revenue generation of the hospitals for a while. Policy is a key concept through which the communities are organized and managed. Most of the policy consequences do not only involve the decisions on why and how to act, but also contain the guidelines on how to allocate the resources to support the implementation of policies and their consequences [7]. Making the right decision requires awareness about the mechanisms that contribute to the efficiency of policy-making. It is not expected that policy-makers, regardless of the factors influencing the policy-making process (including stakeholders), formulate policies for the health system. To face these challenges, taking advantage of the different policy-making theories and patterns in policy analysis helps the policy-makers pass through the superficial layer of policy analysis and reach the deeper layers of the complex health system policies and gain a comprehensive understanding of the target policies [34]. The first and the biggest problem at the beginning of the COVID-19 crisis was the lack of oxygen supply for patients in one of the hospitals. The oxygen generator device was provided by the donors in less than three days. Thus, the cooperation of the donors with the decision-makers and executives of the health system encouraged the medical staff to continue the course of treatment with more strength.

Pan et al. conducted a study based on which a series of multifaceted public health interventions (social distancing, traffic restrictions, staying home, and centralized quarantine) temporarily improved the control of COVID-19 outbreak in Wuhan, China [35]. The government intervened (two-week shutdown) in late February and early March in Iran and asked the people to stay home. However, the citizens of Tehran ignored the warnings and went to the northern cities of the country, especially Babol. This plan could not contribute to breaking the chain of infection. Since no national, provincial or local traffic laws were enacted, movement of the people remained as one of the obvious challenges in controlling the pandemic. In cases of rapid change and fundamental uncertainty, the policy-makers should consider future opportunities. The use of strategic foresight tools is essential for establishing better policies [36]. The policy-making process is rarely a clear procedure. The problem may be identified in the first step, or the policy may never be implemented. The policy-making process is usually very complex. There are many actors, including stakeholders, governmental agencies, political parties, the media and the research community, with different values and goals and different perceptions of reality and policy preferences. Thus, the goal of policy-making is to align different perspectives [37]. This means that policy-making cannot be performed solely by relying on evidence. In fact, none of the political decisions can be determined by evidence alone, because judgments, values and other factors, such as political knowledge and professional experience of different people, are always involved in this process [38, 39].

One of the strengths of the city was the presence of a medical sciences university in it. Therefore, the medical measures started from the very beginning and gradually became more disciplined by formation of the national COVID-19 crisis committee composed of the ICU, lung, heart and internal medicine specialists as well as the infectious disease specialists, virologists, environmental health specialists and epidemiologists. Full prevention, which required the cooperation of different institutions, was not done systematically and remained as one of the weaknesses in dealing with COVID-19. The patients with a positive COVID-19 test who had no indication for hospitalization were released into the society and their contacts could not be traced. Studies have shown that among the few weapons for fighting COVID-19, contact tracing can be the most powerful case [40]. Since the physicians and nurses used to practice according to protocols, in the absence of a specific treatment protocol for COVID-19, they were subjected to moral injury because they did not exactly know, for instance, at what blood oxygen level the patient would require intubation. Although the new treatments and drugs were introduced in different countries at that time, none of them were guaranteed. Therefore, the ICU, pulmonary, internal medicine, infectious and cardiac specialists formed a “committee of critically ill patients” in the hospital for better decision-making. Using the evidences in the world and the country and relying on the experiences they had gained, they established a localized protocol and attempted to manage the treatments and treat the critically ill patients with a higher certainty [41].

One of the important rules in qualitative studies is management of bias. Therefore, the probable effect of the researchers’ bias on the quality of the present study can be mentioned as one of the inherent bias of such studies [42, 43]. In this part of the study, the potential sources of bias from different stages of COVID-19 observational studies were discussed and ways to reduce them were explained. In the first three interviews, the interview guide was sent in advance. It was observed that the participants were ready to act conservatively in answering the questions. We tried not to make the questions visible to the participants beforehand. Therefore, by blinding the participants, performance bias was prevented. Another bias was the selection participant’s bias, participants who were not involved in the development and implementation of COVID-19 programs and policies were excluded from the study, and as a result, there was no escaping selection bias. As there was a selection bias, all findings were reported and thus there were no unreported findings, as a result, reporting bias was avoided. Attrition bias means the loss of participants whose information could affect the results, to compensate, the researchers tried to select participants from different areas of management, treatment and health donors with the right balance.

The limitation in the present study was difficulty in accessing some of the key individuals. Since the interviews were recorded by the researcher, the participants observed the conservatism in their speech, and during the interview, therefore, the researcher was careful not to push the interviewee to defend his or her position. Another limitation of the study was the organizational dependence on (BUMS) for both participants (except health donors) and researchers.

## Conclusions

The COVID-19 policy analysis at the (BUMS) showed that policy-makers and planners were suddenly faced with a large number of patients awaiting treatment. Therefore, most of the decisions, policies and programs were oriented towards taking care and treatment of the patients and providing them with services. The (BUMS) was able to use the capacities and skills of doctors, specialists and experienced nurses to respond to patients waiting for treatment. In this way, it compensated the shortages of financial resources through donors. But it could not use the power of the city government to control traffic and observe health protocols and prevent infection, and there was no explicit program or policy to track the asymptomatic carriers in the society that was the factor, there is no proper connection between the health care systems. Another important factor was the lack of control over the Covid-19 pandemic of irregular and unstable policy formulation and implementation.

## Data Availability

The authors declare that they have no competing interests.

## Abbreviations

COVID-19: Coronavirus Disease
SARS-CoV: Severe Acute Respiratory Syndrome coronavirus
MERS-CoV: Middle East Respiratory Syndrome coronavirus
BiPAP: Bilevel Positive Airway Pressure
CPAP: Continuous Positive Airway Pressure
IVIG: Intravenous Immunoglobulin
ICU: Intensive Care Unit
PCR: Polymerase Chain Reaction
CT scan: Computed Tomography scan
CDC: Centers for Disease Control
NGOs: Non-governmental organizations
WHO: World Health Organization
